# Administration of Repetitive Transcranial Magnetic Stimulation Attenuates A*β*_1-42_-Induced Alzheimer's Disease in Mice by Activating *β*-Catenin Signaling

**DOI:** 10.1155/2019/1431760

**Published:** 2019-03-05

**Authors:** Xueyun Chen, Shu Chen, Weidi Liang, Fang Ba

**Affiliations:** Department of Rehabilitation, Shengjing Hospital of China Medical University, Shenyang 110004, China

## Abstract

Repetitive transcranial magnetic stimulation (rTMS) is a noninvasive and painless technique that has been applied for the treatments of diverse neurodegenerative disorders. In the current study, its anti-Alzheimer's disease (AD) effect was assessed and the mechanism driving the effect was explored. The AD symptoms were induced via the intracranial injection of A*β*_1-42_ in mice and then treated with rTMS of 1 Hz or 10 Hz. The anti-AD effect of rTMS was assessed by Morris water maze (MWM), histological staining and western blotting. The results showed that rTMS administrations of both frequencies improved the cognitive function and suppressed neuron apoptosis in AD mice. Moreover, the treatment also increased the brain BDNF, NGF, and doublecortin levels, which represented the increased viability of neurons by rTMS. The injection of A*β*_1-42_ also increased the expressions of p-GSK-3*β*, p-Tau, and p-*β*-catenin and suppressed the level of total *β*-catenin. After the treatments of rTMS, the level of *β*-catenin was restored, indicating the activation of *β*-catenin signaling. In conclusion, the findings outlined in the current study demonstrated that the anti-AD effect of rTMS was associated with the activation of *β*-catenin, which would promote the survival of neurons.

## 1. Introduction

Alzheimer's disease (AD) is a severe neurodegenerative disorder with progressive neuronal loss, reduced levels of crucial neurotransmitters, and altered synaptic plasticity [1]. During the last decade, the number of AD patient has kept increasing, which brings large burden on the caregivers and the healthcare system [2]. Although great effect has been made to develop disease-modifying therapies for the treatment of AD, conventional pharmacotherapy has thus far failed to slow or reverse the AD process [3]. In this context, the exploration of novel treatment targets for handling AD is not only warranted, but also necessary.

The complicated pathogenesis of AD has limited the development of pharmacotherapy and scientists and clinicians have turned to the effect of nonpharmacotherapies. Thus, transcranial magnetic stimulation (TMS) has drawn a lot of interests for its considerable effect on neurodegenerative disorders [4]. TMS is firstly applied as a noninvasive and painless technique enabling mapping of motor cortical outputs [1]. When the stimulation is applied to the primary motor cortex (M1), the corticospinal pathway and generate motor evoked potential (MEP) are activated in the target muscles [5–7]. With the exploration of the potential of the technique, single pulse stimulation has been gradually replaced by repetitive TMS (rTMS) that consisted of trains of regularly repeating TMS pulses. Compared with single pulse TMS, rTMS temporally summates to cause changes in neural activity that can last for weeks to months even after the treatment [6, 8]. By applying rTMS, researchers have reversed the progression of several neurodegenerative disorders such as Parkinson's disease (PD) [9], AD [10], amyotrophic lateral sclerosis (ALS) [11], and Huntington's disease (HD) [12]. Regarding its effect on AD progression, the study of Cotelli et al. showed that the application of 20 Hz rTMS improved the accuracy of an action naming task in AD patients [13]. In another study performed by the same group, the daily administration of 20 Hz rTMS with 2000 pulses for four weeks showed long-lasting (eight weeks) improvement effect on the language comprehension in moderate AD patients [14]. The previous studies together confirmed the promising potential of rTMS in managing AD. However, even the treatment effect of rTMS on AD has been tested by several studies; the mechanism driving the effect remains partially revealed, which restricts the application of the technique in clinic.

Most recently, it was demonstrated that transgenic mice with suppressed mutant Tau expression showed improved memory function [15, 16]. Moreover, in the study of Li et al., the authors inferred that the hyperphosphorylation of Tau facilitated the function of *β*-catenin, which subsequently promoted the survival of brain neurons [17]. The study also proved that the key mediator between the interaction of Tau and *β*-catenin was GSK-3*β* that phosphorylated both Tau and *β*-catenin [17]. Therefore, the regulation of the activities of GSK-3*β*, Tau, and *β*-catenin might represent a novel treatment strategy of AD. Inspiringly, the previous study showed that magnetic stimulation could influence the expression of presenilin 1 (PS1) that is an upstream regulator of GSK-3*β* [18]. Based on the above information, we hypothesized that the administration of rTMS could attenuate AD by activating *β*-catenin signaling via GSK-3*β* and Tau. To verify the hypothesis, we established AD mice models using A*β*_1-42_ and then assessed the treatment effect of rTMS of two frequencies (1 Hz and 10 Hz) on the cognitive behaviors of the mice. Thereafter, we detected the influence of rTMS administrations on the activities of GSK-3*β*, Tau, and *β*-catenin in the brain tissues of mice to provide some information of the treatment mechanism of rTMS on AD.

## 2. Materials and Methods

### 2.1. Chemicals and Antibodies

A*β*_1-42_ was purchased from GL Biochem (Shanghai) Ltd. (China) and was prepared as oligomers by incubating at a concentration of 1 mg/mL in sterile saline solution, followed by aggregation via incubation at 37°C for 4 days. In Situ Cell Death Detection Kit (11684817910) was purchased from Roche (Switzerland). Enzyme-Linked Immunosorbent Assay (ELISA) Detection Kits for brain derived neurotrophic factor (BDNF) (SEA011Mu) and nerve growth factor (NGF) (SEA105Mu) were purchased from USCN (China). RIPA lysis buffer and (P0013B), BCA Protein Concentration Kit (P0009) and ECL Plus reagent (P0018) were obtained from Beyotime Biotechnology (China). Antibody against GSK-3*β* (24198-1-AP) was purchased from Proteintech (China). Antibodies against phosphorylated GSK-3*β* (p-GSK-3*β*) (#5558), p-*β*-catenin (#9561), and Bax (#2772) were purchased from CST (USA). Antibodies against Tau (ab32057), p-Tau (ab109390), *β*-catenin (ab32572), cleaved caspase 3 (ab49822), Bcl-2 (ab196495), and doublecortin (ab18723) were purchased from Abcam (UK). *β*-actin (bsm-33036M) was purchase from Bioss (China). Secondary IgG-HRP goat anti-rabbit (A0208) and goat anti-murine (A0216) antibodies were purchased from Beyotime Biotechnology (China). Secondary Cy3-labled antibody goat anti-rabbit (A0277) was purchased from Beyotime Biotechnology (China).

### 2.2. Animals and AD Model Induction

C57BL/6 mice (8 weeks old and weighting) were provided by China Medical University and housed at 25±1°C with a humidity of 45-55% with food and water available* ad libitum*. AD model was induced using A*β*_1-42_ injection method. Briefly, mice were anesthetized using 50 mg/kg body weight phenobarbital sodium and then the left striatum of the mouse was exposed. Afterwards, AD model was induced using A*β*_1-42_ according to the previous studies with some modifications [19, 20]: 3 *μ*l A*β*_1-42_ (1 *μ*g/*μ*l) was injected into the left striatum at the following coordinates according to the brain stereotactic map: AP-0.2 mm, ML±1 mm, and DV-2.4 mm. 10 min after the injection, the lesion was sutured and mice were housed routinely. For mice in Sham group, 6-OHDA was replaced by sterile normal saline. Animal surgery protocol was approved by the Institutional Animal Ethics Committee of China Medical University.

### 2.3. Administration of rTMS

For mice treated with rTMS, the treatment was performed one day following the A*β*_1-42_ injection. To explicitly explain the effect of rTMS, two frequencies were set up in the current study. For mice treated with the low frequency (AD+L rTMS), the mice were exposed to low frequency rTMS (1 Hz) with magnetic stimulation intensity set at 30% maximum output (1.26T). The treatment was performed for 14 consecutive days, which consisted of two sessions of rTMS consisting of 1000 pulses in 10 trains. Two sessions were separated by an interval of 2 min to cool down the coil and the interval between each train was 20 s. For mice treated with the high frequency (AD+H rTMS), the frequency was set to 10 Hz.

### 2.4. Tissue Collection

Upon completion of the rTMS treatments, some mice were sacrificed using 150 mg/kg body weight phenobarbital sodium and the hippocampus regions of brain tissues of some mice in each group were collected and preserved at −80°C. The left mice were subjected to Morris Water Maze (MWM) test to determine the effect of rTMS administration on the cognitive function of AD mice.

### 2.5. Morris Water Maze

MWM assays were performed by two investigators blind to the experiment designs. In brief, for visible platform trail in 60 s, rats were allowed to swim for 60 s before getting to the platform for four times each day. If the rats failed, investigator would help the rats to stay on the platform for 10 s before another test. The test included a 1-day probe trial and a 4-day visible platform trial. For probe trial in the 6th day, the platform was removed and then the time and number of each mice spending in the quadrant of the former platform position in 60 s were measured.

### 2.6. Terminal-Deoxynucleotidyl Transferase Mediated Nick End Labeling (TUNEL) Staining

Cell apoptotic rate in brain tissues was determined using TUNEL method: briefly, sections were permeabilized with 0.1% Triton X-100 at room temperature for 8 min and then washed with PBS buffer before incubated in 3% H_2_O_2_ for 10 min at room temperature. After another three 5 min washes using PBS buffer, sections were covered with TUNEL reaction solution and incubated at 37°C for 1 h in dark. The images of the results were captured with a fluorescent microscopy (BX53, OLUMPUS, Japan) at 400× magnification.

### 2.7. Immunochemistry Detection

Brain sections of 5 *μ*m were administrated with alcohol in different concentrations and then washed with PBS for three times. Primaries antibody against doublecortin (1:200) was incubated with the sections at 4°C overnight. Thereafter, secondary Cy3-labled antibodies were incubated with the sections at 37°C for 30 min and then were labeled with horseradish peroxidase-labeled avdin at 37°C for 30 min. After incubation with DAB solution, the sections were subjected to restain using hematoxylin for 3 min and dehydrated using alcohol of different concentrations. Images were captured using a microscope (BX53, Olumpus, Japan) at 400× magnification.

### 2.8. Enzyme-Linked Immunosorbent Assay (ELISA)

The brain levels of BDNF and NGF was detected using ELISA Kits following instructions for manufacturers. The results was represented by OD value at 450 nm as detected by a Microplate Reader (ELX-800, BIOTEK, USA)

### 2.9. Immunofluorescence Detection

The level and distribution of GSK-3*β* in the hippocampus regions of the mice were detected using immunofluorescence detection. Briefly, the sections were firstly permeabilized with 0.5% Triton X-100 for 30 min and then washed with PBS, blocked with 10% goat serum for 15 min, and incubated with primary rabbit polyclonal antibodies against GSK-3*β* overnight at 4°C. Subsequently, the sections were incubated with a fluorescein isothiocyanate-labeled secondary antibody (1:200) for 1 h and restained with 4, 6-diamino-2-phenyl indole (DAPI) for 5 min at room temperature. After three cycles of five-minute washes with PBS, the results were imaged with a fluorescent microscope (BX53, Olumpus, Japan) at 400× magnification.

### 2.10. Western Blotting

Total protein in the brain tissues was lysed using RIPA lysis buffer and collected with centrifugation at 1000 g for 10 min. The concentrations of the protein samples were determined using BCA Protein Concentration Kit and then separated by sodium dodecyl sulfate polyacrylamide gel electrophoresis (SDS-PAGE) at 80V for 2.5 h. The samples were transferred to PVDF membranes and blocked using 5% skim milk powder for 1 h. Primary antibodies against GSK-3*β* (1:1000), p-GSK-3*β* (1:1000), Tau (1:5000), p-Tau (1:10000), *β*-catenin (1:5000), p-*β*-catenin (1:1000), cleaved caspase 3 (1:500), Bcl-2 (1:500), Bax (1:1000), and *β*-actin (1:5000) were incubated with the membranes at 4°C overnight. Afterwards, secondary IgG-HRP antibodies (1:5000) were incubated with the membranes at 37°C for 45 min. Protein bands were developed using ECL Plus reagent and the relative expression levels of proteins were calculated by Gel-Pro-Analyzer (Media Cybernetics, USA).

### 2.11. Statistical Analysis

The data were represented as mean ± standard deviation (SD). One-way analysis of variance and post hoc multiple comparisons using Fisher's Least Significance Difference (LSD) method were performed using GraphPad Prism version 6.0 (GraphPad Software, Inc., San Diego, CA). Statistical significance was accepted when the *P* value (two tailed) is smaller than 0.05.

## 3. Results

### 3.1. Effect of rTMS Treatment on the Cognitive Behaviors of AD Mice

**Figure 1:**
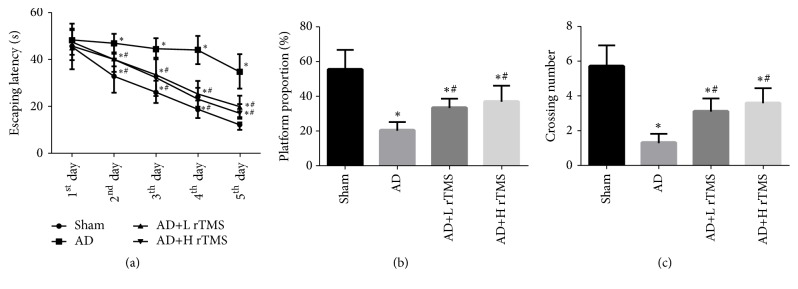
*Effect of rTMS on cognitive function of AD mice*. AD symptoms were induced with intracranial injection of 3 *μ*l A*β*_1-42_ (1 *μ*g/*μ*l) and then treated with rTMS of 1 Hz or 10 Hz. Cognitive function of mice was assessed with MWM assay. (a) Quantitative analysis results of escaping latency. (b) Quantitative analysis results of the platform proportion. (c) Quantitative analysis results of the crossing number. “*∗*” represents statistically significant different from Sham group,* P* < 0.05. “#” represents statistically significant different from AD group,* P* < 0.05. Each assay was represented by six replicates.

The study firstly assessed the effect of rTMS treatment on the cognitive behaviors of mice with MWM test. As shown in Figure 1, the establishment of AD model impaired the cognitive function of mice, which was represented by the increased latency of escaping time (Figure 1(a)), suppressed proportion of penetrating path in platform area (Figure 1(b)), and shortened duration of mice in the platform quadrant (Figure 1(c)). However, for AD mice treated with rTMS of both frequencies, the cognitive function of the mice was significantly improved: the treatment decreased the escaping latency, increased the proportion, and lengthened the staying time (Figure 1). Moreover, compared with the low frequency, the high frequency rTMS treatment showed better improvement effect on the cognitive function, indicating that rTMS treatment could take its action in a frequency-dependent manner (Figure 1).

### 3.2. Effect of rTMS Treatment on the Brain Neuron Viability in AD Mice

**Figure 2:**
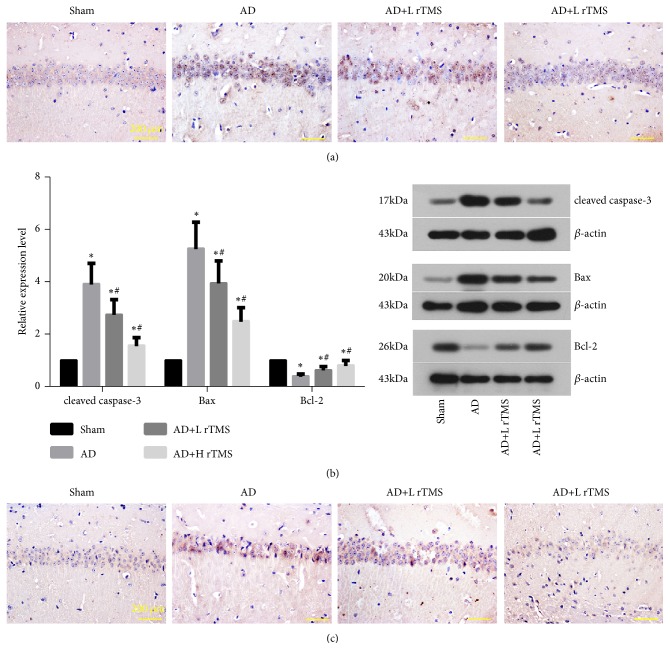
*Effect of rTMS on apoptosis and viability of neurons in hippocampus regions of AD mice*. AD symptoms were induced with intracranial injection of 3 *μ*l A*β*_1-42_ (1 *μ*g/*μ*l) and then treated with rTMS of 1 Hz or 10 Hz. Neuron apoptosis was detected with TUNEL staining and western blotting detection of cleaved caspase-3, Bax, and Bcl-2. Neuron viability was detected with immunochemical detection of doublecortin. (a) Representative images of TUNEL staining. (b) Quantitative analysis results and representative of western blotting detection. (c) Representative images of immunochemical detection of doublecortin. “*∗*” represents statistically significant different from Sham group,* P* < 0.05. “#” represents statistically significant different from AD group,* P* < 0.05. Scale bar, 200 *μ*m. Each assay was represented by six replicates.

The cell apoptosis in the hippocampus region of AD mice was determined with TUNEL staining. The induction of AD symptoms by A*β*_1-42_ increased the number of apoptosis cells (stained blue) in the brain tissues (Figure 2(a)). Associated with the increased apoptosis cell number, the levels of cleaved caspase-3 and Bax were increased while the level of Bcl-2 was suppressed (Figure 2(b)). After the treatments with rTMS, the number of apoptosis cells was dramatically suppressed (Figure 2(a)) and the expression patterns of apoptosis-related members were reversed (Figure 2(b)). Similar to the apoptosis state of neurons, the expression and distribution of doublecortin was firstly increased by A*β*_1-42_ and then suppressed by rTMS treatments (Figure 2(c)), representing the restored viability of neurons in the hippocampus regions.

### 3.3. Effect of rTMS Treatment on the Neurotrophic Factor Levels in AD Mice

**Figure 3:**
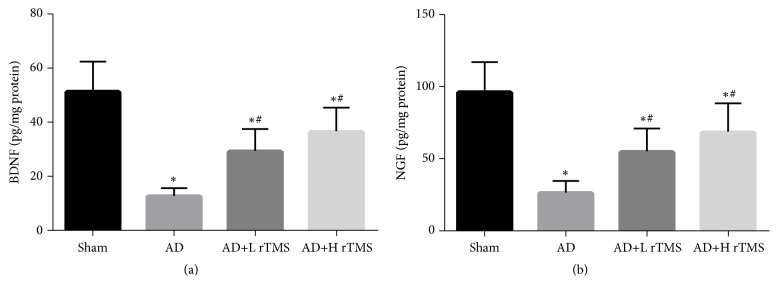
*Effect of rTMS on neurotrophic factor production in AD mice*. AD symptoms were induced with intracranial injection of 3 *μ*l A*β*_1-42_ (1 *μ*g/*μ*l) and then treated with rTMS of 1 Hz or 10 Hz. The production of BDNF and NGF was detected with ELSIA. (a) Quantitative analysis results of ELISA detection of BDNF. (b) Quantitative analysis results of ELISA detection of NGF. “*∗*” represents statistically significant different from Sham group,* P* < 0.05. “#” represents statistically significant different from AD group,* P* < 0.05. Each assay was represented by six replicates.

Except for determining the treatment potential of rTMS by detecting the cognitive function and nerve system integrity of AD mice, we also detected the brain levels of BDNF and NGF that are two classical neurotrophic factors. The results of ELISA detections showed that although the injection of A*β*_1-42_ dramatically suppressed the levels of both factors (Figure 3), the administration of rTMS increased the levels of both indicators (Figure 3). Similar to the effect on cognitive behaviors, the effect of rTMS on neurotrophic factors also increased with frequency (Figure 3).

### 3.4. Effect of rTMS on the Activity of GSK-3*β*, Tau, and *β*-Catenin in AD Mice

**Figure 4:**
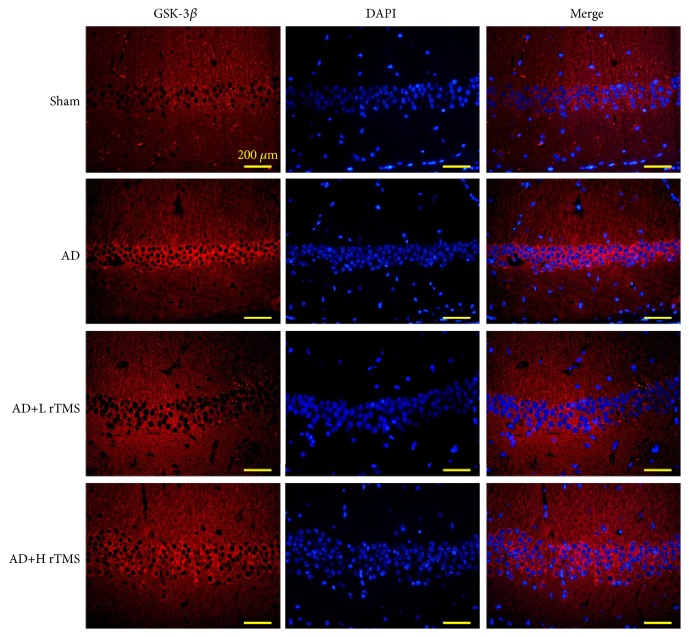
*Effect of rTMS on GSK-3β production and distribution in AD mice*. AD symptoms were induced with intracranial injection of 3 *μ*l A*β*_1-42_ (1 *μ*g/*μ*l) and then treated with rTMS of 1 Hz or 10 Hz. The production and distribution of GSK-3*β* were detected with immunofluorescence detection. Scale bar, 200 *μ*m. Each assay was represented by six replicates.

**Figure 5:**
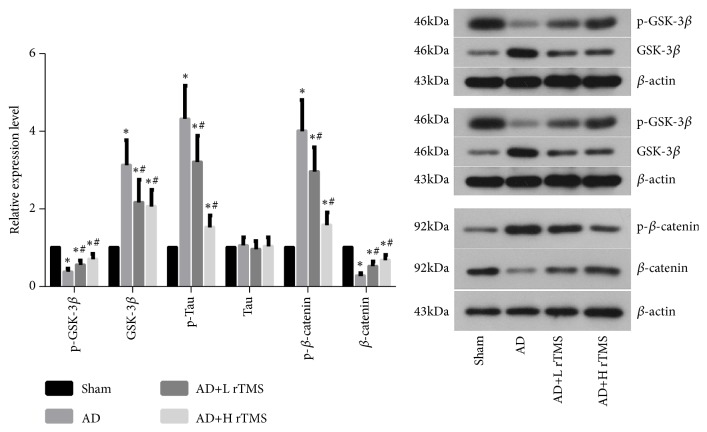
*Effect of rTMS on the activities of GSK-3β, Tau, and β-catenin in AD mice*. AD symptoms were induced with intracranial injection of 3 *μ*l A*β*_1-42_ (1 *μ*g/*μ*l) and then treated with rTMS of 1 Hz or 10 Hz. The activities of GSK-3*β*, Tau, and *β*-catenin were detected with western blotting assay. “*∗*” represents statistically significant different from Sham group,* P* < 0.05. “#” represents statistically significant different from AD group,* P* < 0.05. Each assay was represented by six replicates.

The expressions of GSK-3*β*, Tau, and *β*-catenin were detected with western blotting. It was found that the injection of A*β*_1-42_ increased the protein levels of GSK-3*β* (Figures 4 and 5), p-Tau (Figure 4), and p-*β*-catenin (Figure 4), which would result in the inhibition of prosurvival *β*-catenin/Wnt pathway in brain tissues. After the treatments with rTMS of both frequencies, the tissue levels of all the above three indicators were downregulated while the levels of p-GSK-3*β*, Tau, and *β*-catenin were upregulated (Figure 5), evidently indicating the activation effect of rTMS on *β*-catenin pathways.

## 4. Discussion

The findings outlined in the current study demonstrated that the administrations of rTMS improved the cognitive function of AD mice. Based on the detection of western blotting with brain tissues, it was found that the anti-AD function was associated with the activation of *β*-catenin via the regulation of GSK-3*β* and Tau. However, the phosphorylation of Tau was proved to inhibit the level of *β*-catenin, which was contrary to the results of Li et al. [18]. Moreover, the effect of rTMS on AD symptoms and mice brain molecule levels was exerted in a frequency-dependent manner, implying the application flexibility of the technique.

As a noninvasive stimulation technique on brain, rTMS generates high-intensity magnetic field pulses that are sufficient to cause depolarization of corticospinal tract neurons [21]. The administration of rTMS can affect Ca^2+^ metabolism, cell hydration, and GABA content, which are all critical processes influencing the development and normal function of nerve system. Thus, the technique has been widely employed for the treatment of neurodegenerative disorders such as PD, AD, ALS, and HD [9–12]. In the current study, the anti-AD function of rTMS was tested by MWM assay and histological staining. The injection of A*β*_1-42_ impaired the performance of mice in MWM assay and induced neurons apoptosis in mice brains, which were typical characteristics of the progression of AD. After the treatments of rTMS, the cognitive behaviors and brain structure were restored, confirming the protection effect of rTMS against the AD symptoms [1]. Moreover, the administrations of rTMS also increased the levels of BDNF, NGF, and doublecortin, which also proved the positive effect of rTMS on the viability of neurons [19, 22].

Except for affirming the anti-AD effect of rTMS, we also attempted to provide some preliminary explanations on the treatment mechanism of the technique. Thus, we detected the activities of GSK-3*β*, Tau, and *β*-catenin under the stimulation of rTMS. The factors are also the downstream effectors of PS1 [23], the activity of whom can be influenced by rTMS [18]. In the present study, the results showed that after AD induction, the levels of GSK-3*β*, p-Tau, and p-*β*-catenin were all increased. It has been reported that the phosphorylation of Tau or *β*-catenin is regulated by GSK-3*β* [17]. In the study of Li et al., the authors showed that the hyperphosphorylation of Tau would stabilize the function of *β*-catenin by competitively inhibiting *β*-catenin's phosphorylation by GSK-3*β* [17]. However, the regulating sequence was not supported by our data. The increased phosphorylation of Tau was accompanied by the increased phosphorylation of *β*-catenin. Additionally, the administration of rTMS suppressed the phosphorylation levels of both molecules, which then led to the increased total *β*-catenin level. The results of the current study showed that the hyperphosphorylation of Tau might not be a prerequisite for the stabilization of *β*-catenin. The inhibition of GSK-3*β* would influence the activity of *β*-catenin even with the suppressed phosphorylation level of Tau.

The current study confirmed the anti-AD function of rTMS of low and high frequencies. The effect was associated with the changes in the activities of GSK-3*β*, Tau, and *β*-catenin. However, contrary to the previous studies, the stabilization of *β*-catenin function was not in positive relation to the phosphorylation level of Tau or, in other words, the phosphorylation of Tau was not indispensable for the activation of *β*-catenin signaling and the detail mechanism underlying the conclusion will be explored in the future studies. Collectively, the current study provided a preliminary explanation for the treatment mechanism of rTMS on AD, but to promote the application of the technique in treating neurodegenerative disorders, more comprehensive studies will be needed.

## Data Availability

The data will be provided when required.
